# Complete mitochondrial genomes of two moths in the tribe Trichaeini (Lepidoptera: Crambidae) and their phylogenetic implications

**DOI:** 10.1002/ece3.10188

**Published:** 2023-06-09

**Authors:** Ci Tang, Xicui Du

**Affiliations:** ^1^ College of Plant Protection Southwest University Chongqing China

**Keywords:** Crambidae, mitogenome, phylogeny, Spilomelinae, Trichaeini

## Abstract

The complete mitochondrial genomes of two *Prophantis* species in the tribe Trichaeini (Lepidoptera: Crambidae) were sequenced using high‐throughput sequencing technology. They were assembled and annotated: The complete mitogenomes of *P. octoguttalis* and *P. adusta* were 15,197 and 15,714 bp, respectively, and contain 13 protein‐coding genes (PCGs), 22 transfer RNA genes, two ribosomal RNA genes, and an A + T‐rich region. Their arrangement was consistent with the first sequenced mitogenome of *Bombyx mori* (Bombycidae) in Lepidoptera, which had the *trnM*–*trnI*–*trnQ* rearrangement. The nucleotide composition was obviously AT‐biased, and all PCGs, except for the *cox1* gene (CGA), used ATN as the start codon. Except for *trnS1*, which lacked the DHU stem, all tRNA genes could fold into the clover‐leaf structure. The features of these two mitogenomes were highly consistent with those of other species of Spilomelinae in previous studies. Phylogenetic trees of Crambidae were reconstructed based on mitogenomic data using maximum likelihood and Bayesian inference analysis methods. Results showed that Trichaeini in this study robustly constitute a monophyletic group in Spilomelinae, with the relationships (Trichaeini + Nomophilini) + ((Spilomelini + (Hymeniini + Agroterini)) + Margaroniini). However, the affinities of the six subfamilies Acentropinae, Crambinae, Glaphyriinae, Odontiinae, Schoenobiinae, and Scopariinae within the “non‐PS Clade” in Crambidae remained doubtful with unstable topologies or low supports.

## Graphical abstract

The complete mitochondrial genomes of two *Prophantis* species in the tribe Trichaeini were provided for the first time. Phylogenetic trees of Crambidae were reconstructed based on mitogenomic data using maximum likelihood and Bayesian inference analysis methods. Results showed that Trichaeini in this study robustly constitute a monophyletic group in Spilomelinae, with the relationships (Trichaeini + Nomophilini) + ((Spilomelini + (Hymeniini + Agroterini)) + Margaroniini).

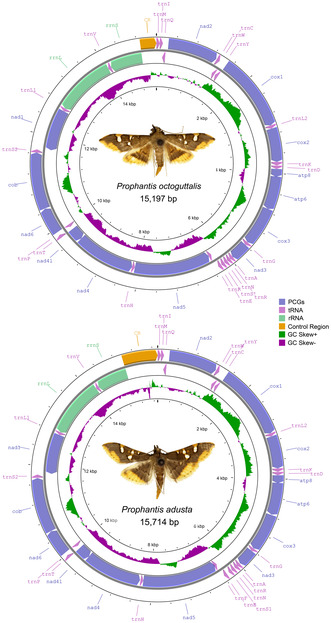

## 1 INTRODUCTION

**FIGURE 1:**
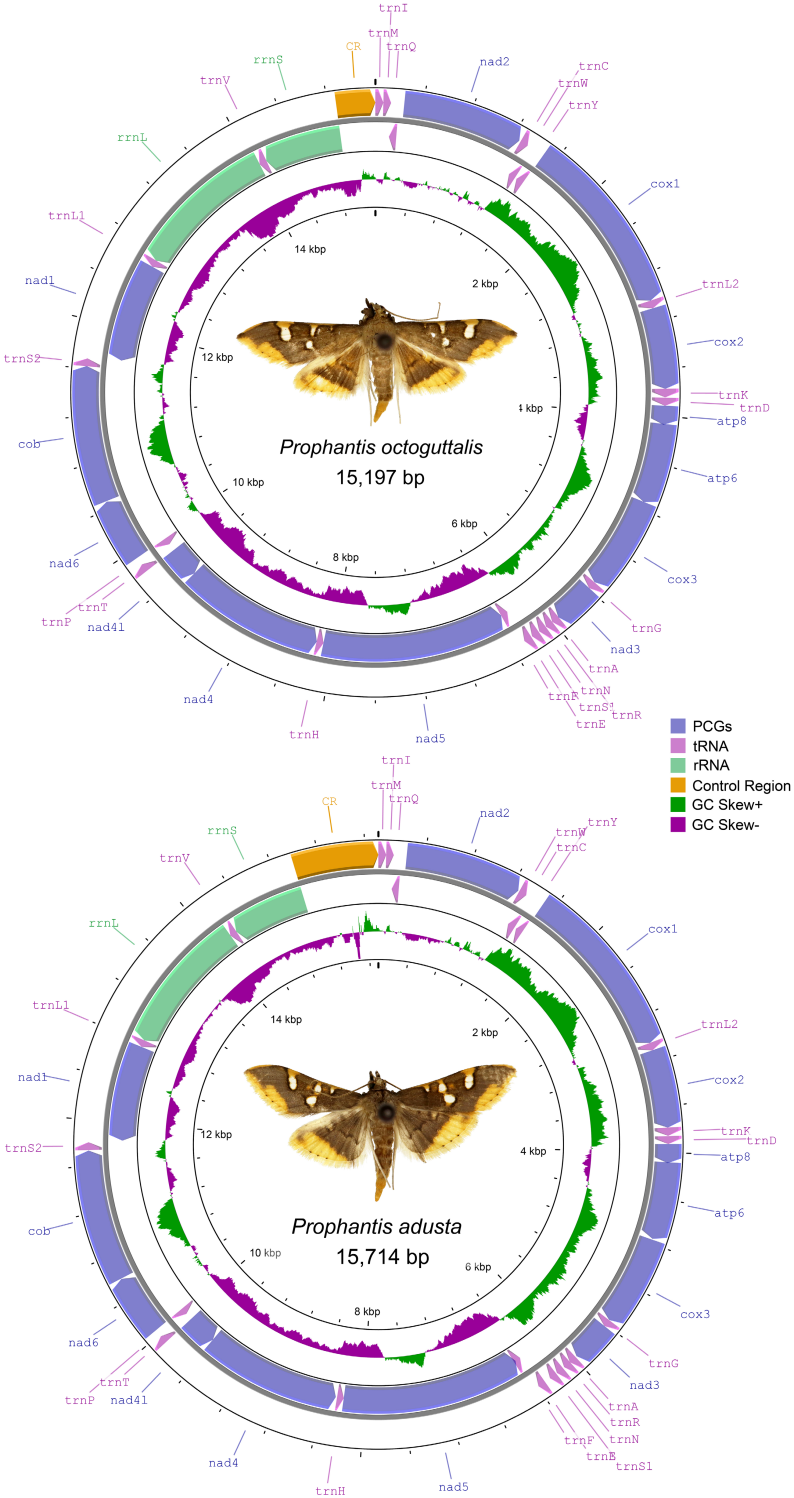
Visualization of the mitochondrial genomes of *Prophantis octoguttalis* and *P. adusta*.

The two *Prophantis* species in the tribe Trichaeini in this study belong to Pyraloidea in Lepidoptera (Figure 1). The Pyraloidea, with more than 16,000 described species worldwide, is one of the largest groups in Lepidoptera, and it is composed of two families: Pyralidae and Crambidae, with Crambidae accounting for 60% of the species diversity (Munroe & Solis, 1999; Nuss et al., 2023). Regier et al. (2012) present a most detailed molecular estimate of relationships to date across the subfamilies of Pyraloidea based on five nuclear genes, in which the Crambidae was divided into three major lineages based on phylogenetic relationships: the “PS clade” (Pyraustinae, Spilomelinae, and Wurthiinae), the “OG clade” (Evergestinae, Glaphyriinae, Noordinae, and Odontiinae), and the “CAMMSS clade” (Acentropinae, Crambinae, Musotiminae, Midilinae, Scopariinae, and Schoenobiinae), forming a system of PS clade + (OG clade + CAMMSS clade). However, there is no stable and convincing phylogenetic relationship within “non‐PS Clade” in the phylogenetic tree topology of the Pyraloidea based on nuclear or mitogenomic data in previous studies (Léger et al., 2021; Liu et al., 2021; Qi et al., 2021; Regier et al., 2012; Yang, Shi, et al., 2018; Zhang et al., 2020). More molecular data, such as the mitogenomes, are in demand to reveal the phylogenetic relationships of the subfamilies in Crambidae.

Spilomelinae is the most species‐rich subfamily in Crambidae, with 4135 described species in 344 genera (Nuss et al., 2023). Currently, a total of 13 tribes in Spilomelinae have been defined by Mally et al. (2019) based on six molecular markers (COI, CAD, EF‐1α, GAPDH, IDH, and RpS5) and 114 adult morphological characters, including Hydririni, Udeini, Lineodini, Wurthiini, Agroterini, Margaroniini, Spilomelini, Herpetogrammatini, Hymeniini, Asciodini, Trichaeini, Steniini, and Nomophilini. Among them, Trichaeini is a tribe with the lowest species richness, with only four genera and 22 species (Nuss et al., 2023). This tribe includes the genus *Prophantis* Warren, 1896, which consists of eight species that have all been poorly studied besides their original descriptions (Warren, 1896). Only *Prophantis octoguttalis* (Felder & Rogenhofer, 1875) and *P. adusta* Inoue, 1986 have been recorded from China. *P. octoguttalis*, the type species of the genus, is widespread, and is mainly distributed in southern China, Australia, India, and the Afrotropical region (Ratnasingham & Hebert, 2007; Wang, 1980). Its larvae feed on *Coffea arabica* (Linnaeus, 1757), and a single larva can harm several berries in succession, which can seriously impact coffee production (Wang, 1980). The adults of *P. adusta* are very similar in appearance to those of *P. octoguttalis* (Figure 1), which makes species identification in these moths very challenging.

The mitochondrial genome (mtDNA) is a closed‐loop DNA double‐helix molecule that varies significantly in length among taxa. The mtDNA of lepidopteran insects is generally 15–16 kb in size and consists of 37 genes, including 13 protein‐coding genes (PCGs), 22 transfer RNA genes (tRNAs), two ribosomal RNA genes (rRNAs), and a control region of variable length also known as A + T‐rich region and D‐loop region (Boore, 1999). Because of its conserved genetic components, compact arrangement, fast evolutionary rate, and maternal inheritance, it contains relevant genetic and developmental information that can be used in phylogenetic studies for different research purposes (Cameron, 2014; Wesley et al., 1979). The mtDNA has been widely used in molecular phylogeny, phylogeography, and genetic differentiation (Heise et al., 1995; Suzuki et al., 2013; Wang et al., 2019).

To date, only 23 mitogenomes of Spilomelinae have been published in GenBank, and no mitogenomes of Trichaeini have been reported. In this study, we sequenced the mitogenomes of *P. octoguttalis* and *P. adusta* of the Trichaeini for the first time, and performed preliminary bioinformatics analysis, including the gene size and arrangement, base composition, codon usage, and tRNA secondary structure, which can help us to understand the features of mitogenomes of Trichaeini and Spilomelinae. To understand the phylogenetic relationships of Spilomelinae and the position of Trichaeini, we reconstructed the phylogenetic tree based on the mitogenomes data of these two species with other available mitogenomes of Crambidae from GenBank by using maximum likelihood (ML) and Bayesian inference (BI) methods. It will provide new perspectives and genomic data for the phylogenetic research in Trichaeini and Spilomelinae.

## 2 MATERIALS AND METHODS

### 2.1 Specimen collection and DNA sequencing

The specimen of *P. octoguttalis* investigated was collected from Wuzhi Mountain in Hainan Province, China, in March 2021; the specimen of *P. adusta* was collected from Fanjing Mountain in Guizhou Province, China, in September 2020. Fresh specimens obtained by light trapping were soaked in anhydrous alcohol and stored at −80°C in the Insect Collection of Southwest University, Chongqing, China. DNA was extracted from the thoracic muscle of each specimen. The mitogenome was sequenced by BGI Genomics on Illumina next‐generation sequencing platform with Read Length of PE150 and Coverage depth of 6 G.

### 2.2 Sequence assembly, annotation, and analysis

The high‐quality data (clean data) of the samples, which were trimmed by BGI Genomics, were saved as fastq. format and imported into analyzed on Geneious Prime v2022.1.1. The published COI sequences of each species (MH418217 and KY370920) were downloaded from GenBank as reference sequences (Lopez‐Vaamonde et al., 2019; Segar et al., 2017), and sequence extension was performed using the “Map to reference” function until repetitive base alignments appeared, indicating that the mitochondrial genome was assembled into a loop.

MAFFT (Multiple Alignment using Fast Fourier Transform) alignment was used to align the reference sequence with the sample sequence, and PCGs were determined based on the similarity between genes. With the help of EditSeq v7.1.0, PCGs were translated into amino acids to further verify the correctness of the start codon, stop codon, and amino acid sequence, to ensure the accuracy of PCGs. The location and secondary structure of tRNA genes were predicted using the MITOS Web Server (Donath et al., 2019), and the chart of secondary structure was mapped using Adobe Illustrator v26.0. Ribosomal RNA genes are relatively conserved and can be determined by the position between the two genes (Boore, 2006). The A + T‐rich region was generally located behind the *rrnL* gene. Mitogenome maps were generated using Proksee (https://proksee.ca/). Sequence length, base composition, gene spacing, and overlap were viewed directly using Geneious Prime v2022.1.1. The base skew was calculated using the formula: AT skew = (A − T)/(A + T) and GC skew = (G − C)/(G + C) (Perna & Kocher, 1995). Relative synonymous codon usage (RSCU) was analyzed using MEGA v10.2.5.

### 2.3 Phylogenetic analysis

A total of 55 mitogenome sequences (two from this study, 53 retrieved from GenBank) were used to construct the phylogenetic tree. The ingroups included five species of Acentropinae, five species of Crambinae, one species of Glaphyriinae, three species of Odontiinae, eight species of Pyraustinae, one species of Schoenobiinae, one species of Scopariinae, and 25 species of Spilomelinae. The four species (*Lista haraldusalis*, *Galleria mellonella*, *Dioryctria yiai*, and *Pyralis farinalis*) of Pyralidae, *Bombyx mori* of Bombycidae, and *Helicoverpa armigera* of Noctuidae were selected as outgroups (Table 1).

**TABLE 1:** Mitochondrial genome sequences used in the phylogenetic analyses.

Family	Subfamily	Species	GenBank ID	References
Bombycidae	Bombycinae	*Bombyx mori*	NC002355	Direct submission
Crambidae	Acentropinae	*Cataclysta lemnata*	MT410858	Direct submission
		*Elophila interruptalis*	KC894961	Park et al. (2014)
		*Parapoynx crisonalis*	KT443883	Direct submission
		*Paracymoriza distinctalis*	KF859965	Ye and You (2016)
		*Paracymoriza prodigalis*	JX144892	Ye et al. (2013)
	Crambinae	*Chilo auricilius*	KJ174087	Cao and Du (2014)
		*Chilo sacchariphagus*	KU188518	Direct submission
		*Chilo suppressalis*	JF339041	Chai et al. (2012)
		*Diatraea saccharalis*	FJ240227	Li et al. (2011)
		*Pseudargyria interruptella*	KP071469	Direct submission
	Glaphyriinae	*Evergestis junctalis*	KP347976	Direct submission
	Odontiinae	*Dausara latiterminalis*	MW732137	Qi et al. (2021)
		*Heortia vitessoides*	NC056800	Qi et al. (2021)
		*Pseudonoorda nigropunctalis*	MW732139	Qi et al. (2021)
	Pyraustinae	*Loxostege aeruginalis*	MN635734	Wu et al. (2022)
		*Loxostege sticticalis*	KR080490	Ma et al. (2016)
		*Loxostege turbidalis*	MN646773	Wu et al. (2022)
		*Ostrinia furnacalis*	NC056248	Li et al. (2020)
		*Ostrinia nubilalis*	NC054270	Fisher et al. (2020)
		*Ostrinia scapulalis*	MT801073	Gschloessl et al. (2020)
		*Ostrinia zealis*	NC048888	Zhou et al. (2020)
		*Pyrausta despicata*	MN956508	Wu et al. (2022)
	Schoenobiinae	*Scirpophaga incertulas*	NC031329	Cao et al. (2014)
	Scopariinae	*Eudonia angustea*	KJ508052	Timmermans et al. (2014)
	Spilomelinae	*Botyodes principalis*	MZ823351	Liu et al. (2021)
		*Cnaphalocrocis medinalis*	JQ305693	Yin et al. (2014)
		*Conogethes pinicolalis*	MT674993	Jeong et al. (2021)
		*Conogethes punctiferalis*	NC021389	Wu et al. (2013)
		*Cydalima perspectalis*	MH602288	Que et al. (2019)
		*Glyphodes pyloalis*	NC025933	Kong and Yang (2016))
		*Glyphodes quadrimaculalis*	KF234079	Park et al. (2015)
		*Haritalodes derogata*	KR233479	Zhao et al. (2016)
		*Marasmia exigua*	MN877384	Zhang et al. (2020)
		*Maruca testulalis*	KJ623250	Zou et al. (2016)
		*Maruca vitrata*	NC024099	Direct submission
		*Nagiella inferior*	MF373813	Direct submission
		*Nomophila noctuella*	KM244688	Tang et al. (2014)
		*Omiodes indicata*	MG770232	Yang, Song, et al. (2018)
		*Palpita hypohomalia*	MG869628	Yang, Shi, et al. (2018)
		*Palpita nigropunctalis*	KX150458	Direct submission
		*Prophantis adusta*	OP559508	This study
		*Prophantis octoguttalis*	OP559507	This study
		*Pycnarmon lactiferalis*	KX426346	Chen et al. (2016)
		*Pycnarmon pantherata*	KX150459	Direct submission
		*Sinomphisa plagialis*	MZ823346	Liu et al. (2021)
		*Spoladea recurvalis*	KJ739310	He et al. (2015)
		*Syllepte taiwanalis*	MZ823348	Liu et al. (2021)
		*Tyspanodes hypsalis*	KM453724	Wang et al. (2016)
		*Tyspanodes striata*	KP347977	Direct submission
Noctuidae	Heliothinae	*Helicoverpa armigera*	NC014668	Yin et al. (2010)
Pyralidae	Epipaschiinae	*Lista haraldusalis*	KF709449	Ye et al. (2015)
	Galleriinae	*Galleria mellonella*	KT750964	Park et al. (2017)
	Phycitinae	*Dioryctria yiai*	MN658208	Wu et al. (2020)
	Pyralinae	*Pyralis farinalis*	MN442120	Mao et al. (2019)

We used two datasets: (1) PCG123: all three codon positions of 13 PCGs; (2) PCG123RT: all three codon positions of 13 PCGs, two rRNA genes and 22 tRNA genes. ML and BI were used to construct phylogenetic trees.

ModelFinder (Kalyaanamoorthy et al., 2017) was used to partition the data based on Bayesian Information Criterion BIC and find the best partitioning scheme and base substitution models for ML and BI. ML was analyzed using IQ‐TREE v1.6.8 (Minh et al., 2013; Nguyen et al., 2015), with the standard bootstrap of 1000 replications; bootstrap values (BS) ≥ 70% were considered to represent moderate confidence. BI was performed under MrBayes v3.2.6, with the following parameters: two independent runs, each with four independent Markov Chain Monte Carlo runs, including three heated chains and one cold chain, were set to run for 1 × 10^7^ generations, with simultaneous sampling every 1000 generations. The initial 25% of the sampled trees were discarded as burn‐ins. Chain convergence was assumed when the mean standard deviation of the split frequencies fell below 0.01. Bayesian posterior probability, in which the support of each node of the BI tree was greater than or equal to 0.95, was considered high confidence. The phylogenetic tree was constructed using Figtree v.1.4.4.

## 3 RESULTS AND DISCUSSION

### 3.1 Basic structure

The mitochondrial genomes of *P. octoguttalis* and *P. adusta* both include 37 genes and one control region (Figure 1). Four PCGs (*nad1*, *nad4*, *nad5*, and *nad4l*), two rRNA genes (*rrnL* and *rrnS*), and eight tRNA genes (*trnQ*, *trnC*, *trnY*, *trnF*, *trnH*, *trnP*, *trnL1*, and *trnV*) are encoded from the minority strands. The remaining 23 genes are encoded from the majority of the strands (Table 2). The full length of the mitochondrial genomes of *P. octoguttalis* is 15,197 bp, and *P. adusta* has the longest mitochondrial genome length of 15,719 bp among sequenced species of Spilomelinae. There are eight gene overlaps and 15 gene gaps in the mitogenome of *P. octoguttalis*, while five gene overlaps and 18 gene gaps are found in the mitogenome of *P. adusta*. Except for the original arrangement of the insect mitogenome, the *trnM*–*trnI*–*trnQ* rearrangement occurs in both species, which is identical to that of the other sequenced Spilomelinae and the Lepidopteran model organism, *Bombyx mori* (Linnaeus, 1758) (Dai et al., 2013).

**TABLE 2:** Mitogenomic organization of *Prophantis octoguttalis* and *P. adusta.*

Gene	Strand	Position		Size		Intergenic nucleotides		Start/stop codon		Anticodon
		Po	Pa	Po	Pa	Po	Pa	Po	Pa	
*trnM*	J	1–67	1–68	67	68	0	0			CAT
*trnI*	J	68–131	69–133	64	65	−3	‐3			GAT
*trnQ*	N	129–197	131–199	69	69	45	46			TTG
*nad2*	J	243–1256	246–1259	1014	1014	13	11	ATT/TAA	ATT/TAA	
*trnW*	J	1270–1337	1271–1338	68	68	−8	−8			TCA
*trnC*	N	1330–1394	1331–1400	65	70	19	20			GCA
*trnY*	N	1414–1482	1421–1487	69	67	8	15			GTA
*cox1*	J	1491–3021	1503–3033	1531	1531	0	0	CGA/T‐	CGA/T‐	
*trnL2*	J	3022–3088	3034–3100	67	67	0	0			TAA
*cox2*	J	3089–3770	3101–3782	682	682	0	0	ATG/T‐	ATG/T‐	
*trnK*	J	3771–3841	3783–3853	71	71	3	3			CTT
*trnD*	J	3845–3911	3857–3924	67	68	0	0			GTC
*atp8*	J	3912–4070	3925–4089	159	165	−7	−7	ATA/TAA	ATA/TAA	
*atp6*	J	4064–4738	4083–4757	675	675	−1	8	ATG/TAA	ATG/TAA	
*cox3*	J	4738–5526	4766–5554	789	789	2	2	ATG/TAA	ATG/TAA	
*trnG*	J	5529–5593	5557–5621	65	65	0	0			TCC
*nad3*	J	5594–5947	5622–5975	354	354	−1	12	ATA/TAA	ATT/TAA	
*trnA*	J	5947–6011	5988–6053	65	66	1	−1			TGC
*trnR*	J	6013–6076	6053–6118	64	66	4	14			TCG
*trnN*	J	6081–6145	6113–6198	65	66	7	9			GTT
*trnS1*	J	6153–6218	6208–6273	66	66	9	54			GCT
*trnE*	J	6228–6293	6328–6394	66	67	−2	−2			TTC
*trnF*	N	6292–6358	6393–6462	67	70	0	0			GAA
*nad5*	N	6359–8093	6463–8197	1735	1735	0	0	ATT/T‐	ATT/T‐	
*trnH*	N	8094–8159	8198–8263	66	66	−1	13			GTG
*nad4*	N	8159–9499	8277–9617	1341	1341	0	0	ATG/TAA	ATG/TAA	
*nad4l*	N	9500–9793	9618–9911	294	294	2	2	ATG/TAA	ATG/TAA	
*trnT*	J	9796–9862	9914–9979	67	66	0	0			TGT
*trnP*	N	9863–9928	9980–10,045	66	66	2	2			TGG
*nad6*	J	9931–10,464	10,048–10,581	534	534	5	4	ATT/TAA	ATT/TAA	
*cob*	J	10,470–11,618	10,586–11,734	1149	1149	−1	5	ATG/TAA	ATG/TAA	
*trnS2*	J	11,618–11,682	11,740–11,806	65	67	18	19			TGA
*nad1*	N	11,701–12,639	11,826–12,764	939	939	0	1	ATG/TAA	ATG/TAA	
*trnL1*	N	12,640–12,707	12,766–12,833	68	68	29	0			TAG
*rrnL*	N	12,708–14,062	12,834–14,174	1355	1341	0	0			
*trnV*	N	14,063–14,133	14,175–14,243	71	69	0	0			TAC
*rrnS*	N	14,134–14,870	14,244–14,979	737	736	0	0			
CR		14,871–15,197	14,980–15,714	327	735					

The mitogenome sequences of both species show obvious AT biases. The nucleotide content of the *P. octoguttalis* mitogenome is A: 41.0%, T: 40.5%, C: 11.0%, and G: 7.5%, and the *P. adusta* mitogenome is A: 40.8%, T: 40.7%, C: 11.0%, and G: 7.4%. The AT content is 81.5% and 81.6%, respectively, which is much higher than the GC content. The AT skew is 0.006 and 0.001, and the GC skew is −0.189 and −0.196, respectively, showing a slight A skew and a significant C skew (Table 3). However, the mitogenomes of other sequenced Spilomelinae are biased toward T and C, showing negative AT skew and negative GC skew.

**TABLE 3:** Nucleotide composition of *Prophantis octoguttalis* and *P. adusta.*

Regions	T%		C%		A%		G%		A + T%		AT skew		GC skew		Total (bp)	
	Po	Pa	Po	Pa	Po	Pa	Po	Pa	Po	Pa	Po	Pa	Po	Pa	Po	Pa
Whole	40.5	40.7	11.0	11.0	41.0	40.8	7.5	7.4	81.5	81.6	0.006	0.001	−0.189	−0.196	15,197	15,714
PGCs	39.8	39.7	11.6	12.1	40.5	39.9	8.2	8.4	80.3	79.6	0.01	0.003	−0.173	−0.181	11,196	11,219
1st codon	41.6	35.2	9.0	14.4	41.1	37.9	8.4	12.5	82.7	73.1	−0.006	0.037	−0.034	−0.071	3732	3740
2st codon	36.8	42.5	14.6	13.4	38.1	37.3	10.5	6.7	74.9	79.8	0.017	−0.065	−0.163	−0.333	3732	3740
3st codon	41.0	41.4	11.1	8.4	42.2	44.4	5.7	5.8	83.2	85.8	0.014	0.035	−0.321	−0.183	3732	3739
rRNA	42.4	43.3	10.1	9.7	42.5	42.0	5.0	5.0	84.9	85.3	0.001	−0.015	−0.338	−0.32	2092	2077
tRNA	40.7	39.8	9.9	10.3	41.4	42.1	7.9	7.7	82.2	82.0	0.009	0.028	−0.112	−0.144	1468	1481
RNAs	41.7	41.9	10.0	10.0	42.1	42.0	6.2	6.1	83.8	83.9	0.005	0.001	−0.235	−0.242	3560	3558
CR	49.8	49.7	3.1	2.2	46.2	47.1	0.9	1.1	96.0	96.7	−0.038	−0.027	−0.55	−0.333	327	735

### 3.2 PCGs and codon usage

Thirteen PCGs are identified in the mitogenomes of *P. octoguttalis* and *P. adusta*. Among them, *atp8*, *atp6*, *cox1*, *cox2*, *cox3*, *nad2*, *nad3*, *nad6*, and *cytb* are encoded by the majority strand, and the remaining four genes are encoded by the minority strand. In *P. octoguttalis*, there are two overlaps, a 7 bp overlap between *atp8* and *atp6* and 1 bp overlap between *atp6* and *cox3*. In *P. adusta*, there is only a 7 bp overlap between *atp8* and *atp6*. As with most other Spilomelinae, the start codons of all genes are typical ATN, except for *cox1*, whose start codon is CGA. The stop codons of *cox1*, *cox2*, and *nad5* are terminated by an incomplete stop codon T, and the remaining genes are terminated by TAA, which is the most frequent stop codon, although termination coding by TAG has been reported in a few sequenced Spilomelinae. Among the PCGs, the AT content is 80.3% and 79.6%, respectively. The AT bias of these two species is more significant in the third codon, and the AT content of the third codon (83.2% and 85.8%) is higher than that of the first (73.1% and 82.7%) and second codons (74.9% and 79.8%). The AT skew of these two species is 0.01 and 0.003, and their GC skew is −0.173 and −0.181, respectively, showing a slight A skew and an obvious C skew.

**FIGURE 2:**
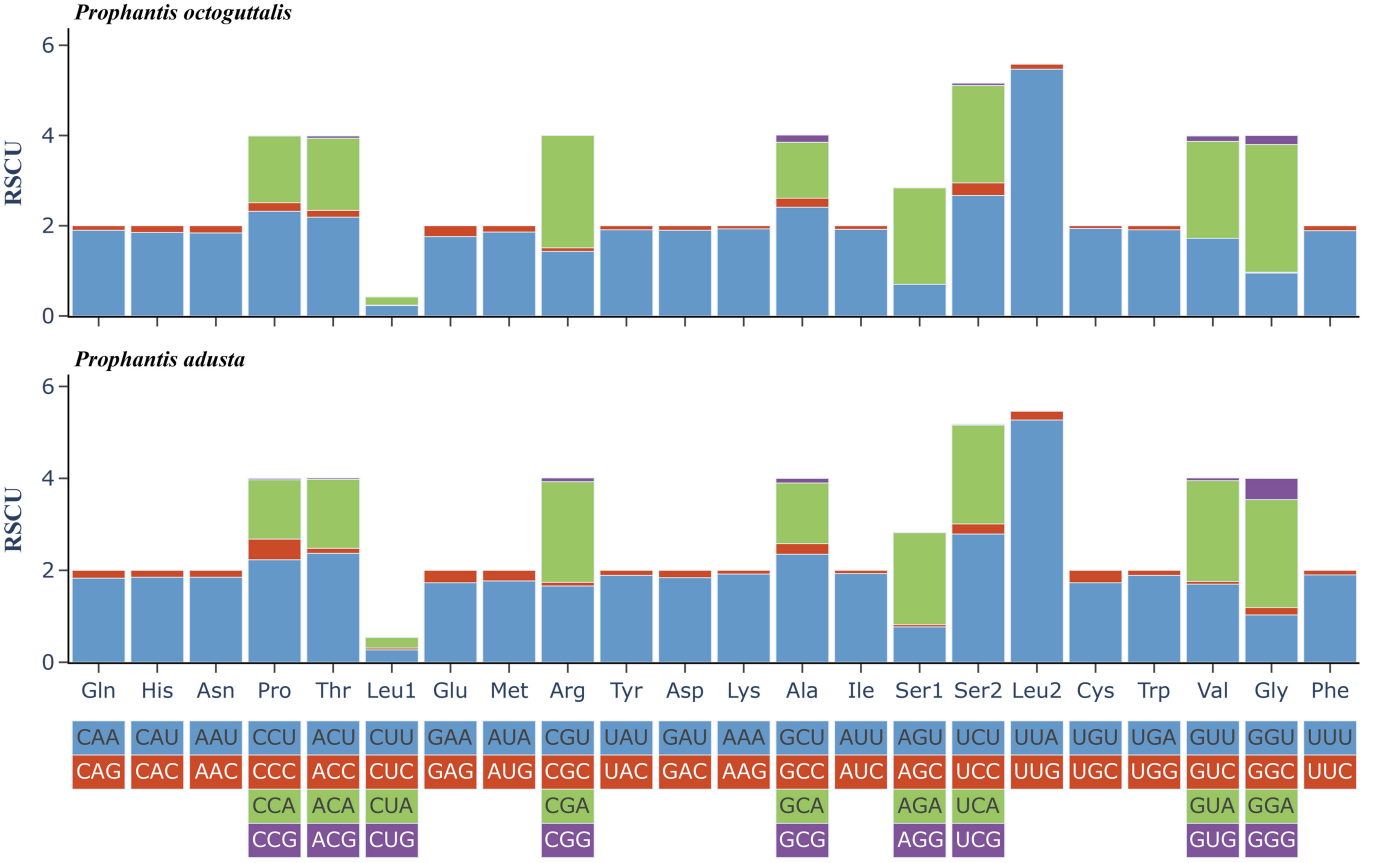
Relative synonymous codon usage (RSCU) of *Prophantis octoguttalis* and *P. adusta*.

The concatenated lengths of the 13 PCGs of *P. octoguttalis* and *P. adusta* are 11,196 and 11,219 bp, encoding 3721 and 3728 amino acids, respectively. Statistics on the RSCU of *P. octoguttalis* and *P. adusta* show that the codons UUA(L), AUU(I), UUU(F), AUA(M), and AAU(N) are used most frequently. In *P. octoguttalis*, CUG, GUC, CCG, CGG, AGC, and AGG do not participate in amino acid synthesis, while in *P. adusta*, CUG and AGG do not participate. The codons of amino acids with RSCU >1 all contain A or U (Figure 2), and the preference of these codons indirectly reflects the AT skew.

### 3.3 rRNA genes and tRNA genes

In the mitogenomes of *P. octoguttalis* and *P. adusta*, two rRNA genes are encoded by the minority strand, with concatenated lengths of 2092 and 2077 bp, respectively. The *rrnL* gene is located between the *trnL1* and *trnV* genes, which are 1355 and 1341 bp long, respectively; the *rrnS* gene is located between the *trnV* gene and the A + T‐enriched regions, which are 737 and 736 bp long, respectively.

**FIGURE 3:**
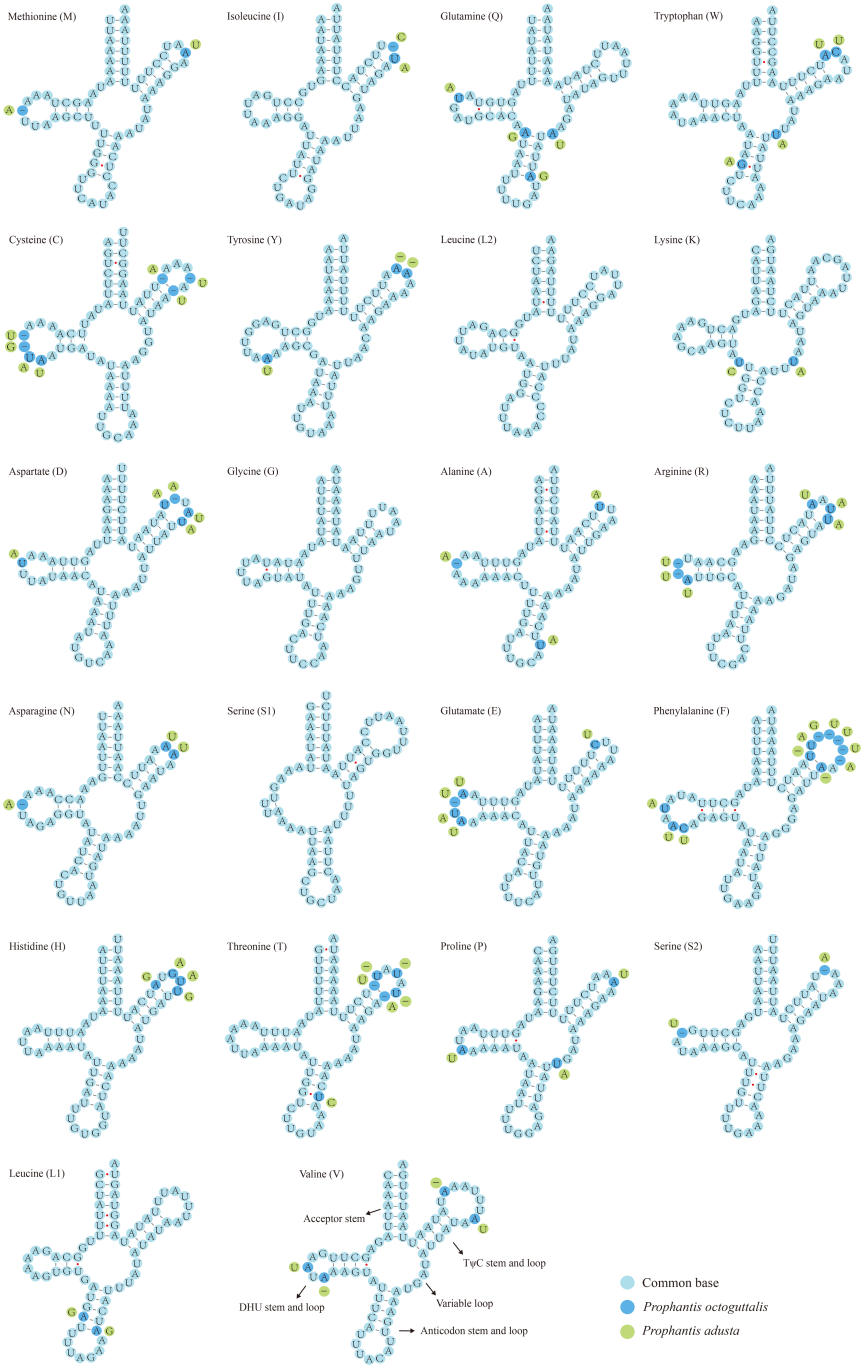
Secondary structure of tRNA of *Prophantis octoguttalis* and *P. adusta*.

In the mitogenomes of these two species, there are 22 tRNA genes with concatenated lengths of 1468 and 1481 bp, respectively. A total of 14 genes (*trnM*, *trnI*, *trnW*, *trnL2*, *trnK*, *trnD*, *trnG*, *trnA*, *trnR*, *trnN*, *trnS1*, *trnE*, *trnT*, and *trnS2*) are encoded by the majority chain, and the remaining eight genes are encoded by the minority chain, with the length of each gene ranging from 64 bp (*P. octoguttalis*) to 71 bp. Except for *trnS1* (AGN), which lacks the DHU stem, the secondary structures of the remaining 21 tRNAs fold into a typical clover‐leaf structure (Figure 3). As with other sequenced Spilomelinae, there are G‐U and U–U base mismatches in the tRNA genes, which mostly occur in the DHU, AA acceptor, and anticodon stems, the G‐U mismatch of *trnS1*(AGN) gene occurs in the TψC stem.

The AT content of the RNA gene of these two species is more than 80%, showing an obvious AT bias. As for base skew, both species show a slight A skew and an obvious C skew.

### 3.4 Non‐coding regions

The mitogenome of *P. octoguttalis* has eight gene overlaps totaling 24 bp, with a maximum overlap length of 8 bp between the *trnW* and *trnC* genes, and 15 gene spacings totaling 172 bp, with a maximum spacing length of 45 bp between the *trnQ* and *nad2* genes. The mitogenome of *P. adusta* has five gene overlaps totaling 21 bp, with a maximum overlap length of 8 bp between the *trnW* and *trnC* genes, and 18 gene spacings totaling 240 bp, with a maximum spacing length of 54 bp between the *trnS1* and *trnE* genes.

The control region of the mitogenome of these two species is located between the *rrnS* and *trnM* genes, with full lengths of 327 and 735 bp, respectively. Both sequences show a clear AT bias, with an AT content of 96.0% and 96.7%, respectively, which is significantly higher than that of GC. The AT skew and GC skew of both sequences are negative, showing a slight T skew and an obvious C skew.

### 3.5 Phylogenetic relationships

**FIGURE 4:**
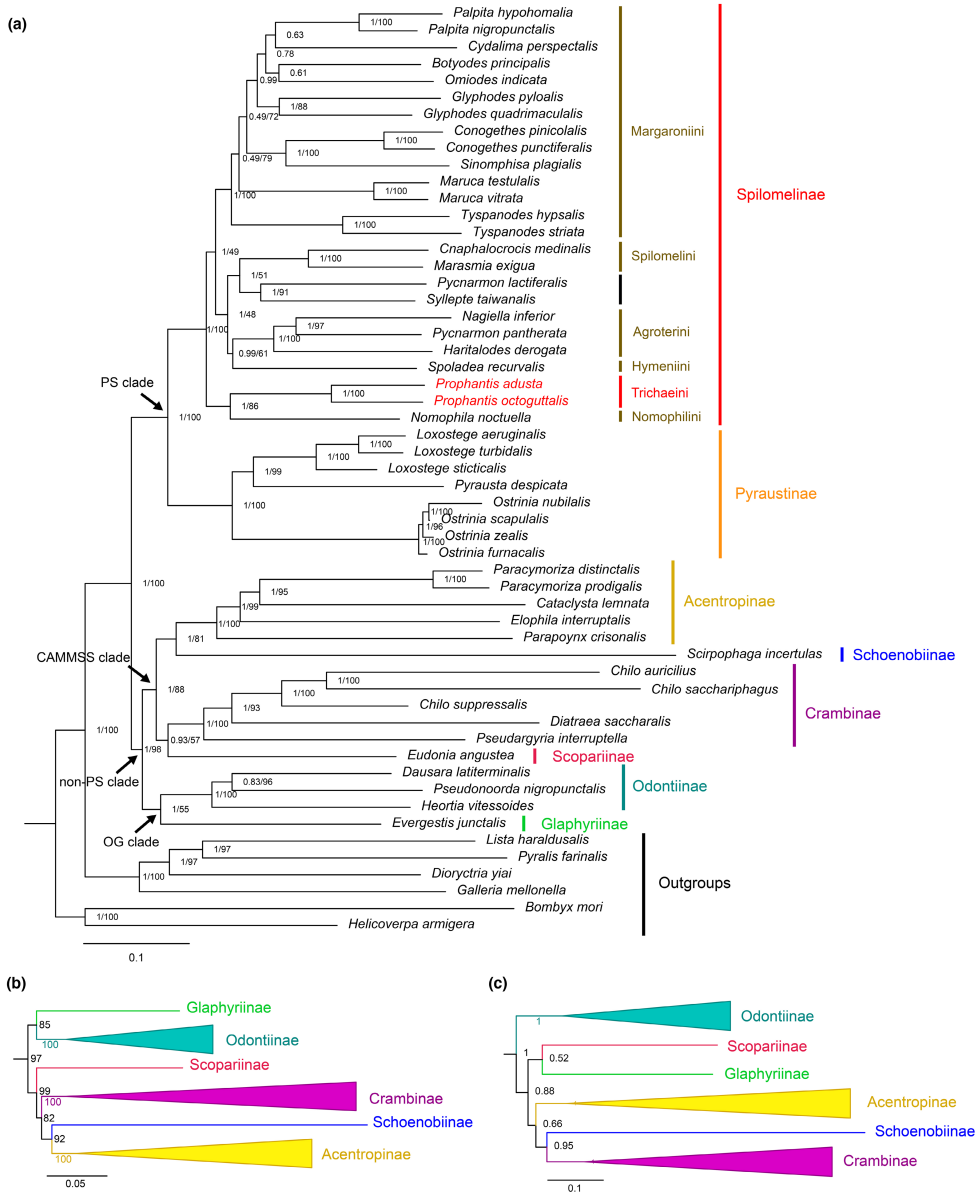
Phylogenetic tree constructed with Bayesian inference (BI) and maximum likelihood (ML) analyses based on two datasets show the similar topology except for the “non‐PS clade”: (a) the BI tree of dataset PCG123RT and the ML tree of dataset PCG123. (b) the “non‐PS clade” of ML tree of dataset PCG123RT. (c) the “non‐PS clade” of BI tree of dataset PCG123. The values around the nodes are posterior probability and bootstrap support.

Four phylogenetic trees of Crambidae were reconstructed using ML and BI analyses based on two datasets: PCG123 and PCG123RT (Figure 4). All phylogenetic trees recovered the monophyly of Crambidae with strong support (PP = 1/BS = 100). The eight subfamilies of Crambidae in all phylogenetic trees were divided into two major sister lineages, the “PS clade” and the “non‐PS clade,” which were first defined by Regier et al. (2012). Spilomelinae and Pyraustinae were sister groups to each other (PP = 1/BS = 100), forming the “PS clade,” which was consistent with previous studies based on molecular data (Léger et al., 2021; Regier et al., 2012) or mitogenomic data (Jeong et al., 2021; Liu et al., 2021; Qi et al., 2021; Yang, Shi, et al., 2018; Zhang et al., 2020).

In Spilomelinae, all phylogenetic results showed that the monophyly of Trichaeini was well supported (PP = 1/BS = 100), and the relationships within Spilomelinae were (Trichaeini + Nomophilini) + ((Spilomelini + (Hymeniini + Agroterini)) + Margaroniini). With the exception of the newly sequenced species of Trichaeini, the phylogenetic relationship among the tribes was roughly consistent with Liu et al. (2021) with Agroterini, Hymeniini, Margaroniini, and Spilomelini grouped into one branch, sister to Nomophilini. Our results showed that Trichaeini and Nomophilini were related to each other as a sister group (PP = 1/BS = 86) and were first separated from the base of the subfamily Spilomelinae. This confirmed the results of Matsui et al. (2022) based on one mitochondrial gene (COI) and three nuclear genes (CAD, EF‐1α, and RpS5). However, in the phylogenetic tree in Mally et al. (2019) based on one mitochondrial gene (COI) and five nuclear genes (CAD, EF‐1α, RpS5, GAPDH, and IDH), Trichaeini and (Steniini + Nomophilini) formed a sister group relationship, which was inconsistent with (Trichaeini + Nomophilini) + Steniini in Matsui et al. (2022). Therefore, more samples, especially those of the closely related species of Steniini and Nomophilini, are expected to be sequenced for the complete mitochondrial genomes in the future research, in order to clarify the phylogenetic relationships among these three tribes.

The differences among the four phylogenetic trees constructed in this study were mainly concentrated in the “non‐PS clade.” The “non‐PS clade” was divided into the “OG clade” and the “CAMMSS clade” (PP = 1/BS = 98) in the BI tree of dataset PCG123RT and the ML tree of dataset PCG123. The “OG clade” consisted of Glaphyriinae and Odontiinae, which were related to each other as sister groups, with a high to low support (PP = 1/BS = 55) and the monophyly of Odontiinae was highly supported (PP = 1/BS = 100). Acentropinae, Crambinae, Schoenobiinae, and Scopariinae formed the “CAMMSS clade,” which presented two close relationships, Acentropinae and Schoenobiinae as sister group (PP = 1/BS = 81), Scopariinae and Crambinae as sister group with a high to low support (PP = 0.93/BS = 57). This was consistent with the results in previous studies based on nuclear and mitochondrial markers (Léger et al., 2021; Regier et al., 2012) and mitochondrial genome (Jeong et al., 2021; Liu et al., 2021; Qi et al., 2021). The affinities of the subfamilies in the “CAMMSS clade,” which based on the ML tree of dataset PCG123RT in this study, were exhibited different topologies: Scopariinae + (Crambinae + (Acentropinae + Schoenobiinae)), which was consistent with the ML tree of dataset PCG123 and PCG123RT in Liu et al. (2021). In the BI tree of dataset PCG123, the phylogenetic relationship of the “non‐PS clade” was: Odontiinae + ((Scopariinae + Glaphyriinae) + (Acentropinae + (Schoenobiinae + Crambinae))), with low support, which was completely different from the above situation. The phylogenetic topology varies among the subfamilies within the “non‐PS clade” in different datasets, probably due to only one sample each in Schoenobiinae, Scopariinae, and Glaphyriinae, thus causing a long branch attraction.

On the basis of the above analyses, our analyses confirmed the sister relationship of Pyraustinae and Spilomelinae with strong support. Trichaeini in this study robustly constitute a monophyletic group in Spilomelinae, with the relationships (Trichaeini + Nomophilini) + ((Spilomelini + (Hymeniini + Agroterini)) + Margaroniini). Within the “non‐PS clade,” the monophyly of Acentropinae, Crambinae, and Odontiinae was well supported. The close relationship between Odontiinae and Glaphyriinae, between Schoenobiinae and Acentropinae, and between Scopariinae and Crambinae seemed to be more realistic.

## 4 CONCLUSIONS

In this study, we reported the complete mitogenomes of two *Prophantis* species, *P. octoguttalis* and *P. adusta*, belonging to the tribe Trichaeini, for the first time, and analyzed their gene size and arrangement, base composition, codon usage, and tRNA secondary structure, etc., which were highly consistent with those of other previously studied species of Spilomelinae. The two mitogenomes were typical of Lepidopteran insects. Combined with the published mitogenome sequences of Crambidae, all phylogenetic trees based on the different datasets confirmed the monophyly and position of Trichaeini and showed satisfactorily high support values. However, its sister group was not completely resolved, combined with previous multisite studies. In addition, the phylogenetic relationships within Crambidae in phylogenetic tree in our present study were in general agreement with previous studies, whereas the affinities in the “non‐PS clade” were still unstable and require further investigation. Therefore, improving sample coverage and combining different molecular markers, such as mitochondrial genome and nuclear genes, should be considered in the future research on these taxa.

## AUTHOR CONTRIBUTIONS

**Ci Tang:** Conceptualization (equal); data curation (equal); formal analysis (lead); methodology (lead); software (lead); writing – original draft (lead); writing – review and editing (equal). **Xicui Du:** Conceptualization (equal); data curation (equal); funding acquisition (lead); project administration (lead); resources (lead); supervision (lead); writing – review and editing (equal).

## FUNDING INFORMATION

This work was sponsored by the National Natural Science Foundation of China (31772500) and by the Natural Science Foundation of Chongqing, China (No. CSTB2022NSCQ‐MSX1164).

## CONFLICT OF INTEREST STATEMENT

All authors declare no conflicts of interest.

## Data Availability

The complete mitogenomes of two *Prophantis* species in this study are deposited in GenBank of NCBI under accession number OP559507 (*P. octoguttalis*) and OP559508 (*P. adusta*).
